# Status of Emergency Obstetric Care in Six Developing Countries Five Years before the MDG Targets for Maternal and Newborn Health

**DOI:** 10.1371/journal.pone.0049938

**Published:** 2012-12-06

**Authors:** Charles Ameh, Sia Msuya, Jan Hofman, Joanna Raven, Matthews Mathai, Nynke van den Broek

**Affiliations:** 1 Maternal and Newborn Health Unit, Liverpool School of Tropical Medicine, Liverpool, United Kingdom; 2 Department of Maternal, Newborn, Child and Adolescent Health, World Health Organisation, Geneva, Switzerland; Université de Montréal, Canada

## Abstract

**Background:**

Ensuring women have access to good quality Emergency Obstetric Care (EOC) is a key strategy to reducing maternal and newborn deaths. Minimum coverage rates are expected to be 1 Comprehensive (CEOC) and 4 Basic EOC (BEOC) facilities per 500,000 population.

**Methods and Findings:**

A cross-sectional survey of 378 health facilities was conducted in Kenya, Malawi, Sierra Leone, Nigeria, Bangladesh and India between 2009 and 2011. This included 160 facilities designated to provide CEOC and 218 designated to provide BEOC. Fewer than 1 in 4 facilities aiming to provide CEOC were able to offer the nine required signal functions of CEOC (23.1%) and only 2.3% of health facilities expected to provide BEOC provided all seven signal functions. The two signal functions least likely to be provided included assisted delivery (17.5%) and manual vacuum aspiration (42.3%). Population indicators were assessed for 31 districts (total population = 15.7 million). The total number of available facilities (283) designated to provide EOC for this population exceeded the number required (158) a ratio of 1.8. However, none of the districts assessed met minimum UN coverage rates for EOC. The population based Caesarean Section rate was estimated to be <2%, the maternal Case Fatality Rate (CFR) for obstetric complications ranged from 2.0–9.3% and still birth (SB) rates ranged from 1.9–6.8%.

**Conclusions:**

Availability of EOC is well below minimum UN target coverage levels. Health facilities in the surveyed countries do not currently have the capacity to adequately respond to and manage women with obstetric complications. To achieve MDG 5 by 2015, there is a need to ensure that the full range of signal functions are available in health facilities designated to provide CEOC or BEOC and improve the quality of services provided so that CFR and SB rates decline.

## Introduction

Sub Saharan Africa (SSA) and South Asia contribute to 87% of the estimated 358,000 maternal deaths and more than three-quarters of the 3.6 million neonatal deaths occurring each year globally [1], [2]. Agreed strategies to address this include ensuring Skilled Birth Attendance (SBA) and Emergency Obstetric Care (EOC) are available and accessible [3]–[5]. The availability of EOC depends on having in place a set of seven key interventions known as “signal functions” for health facilities providing Basic EOC (BEOC) and nine for facilities providing Comprehensive EOC (CEOC) (Table 1) [6]. It has been estimated that with EOC in place, up to 60% of maternal deaths, 40% of intrapartum-related neonatal deaths and 45–75% of intra-partum stillbirths could be averted [2], [6]–[8].

**Table 1:** Signal functions for Essential (or Emergency) Obstetric Care.

Basic EOC services	Comprehensive EOC services
iv/im antibiotics	All included in Basic EOC (1–7) plus:
iv/im oxytocic drugs	Caesarean Section
iv/im anticonvulsants	Blood Transfusion
Removal of retained products of conception(e.g. by manual vacuum aspiration)	
Assisted vaginal delivery (usually ventouse delivery)	
Resuscitation of the newborn baby using a bag and mask	

**Source:** WHO 2009: Managing emergency obstetric care: a handbook.

Availability, utilization and quality of EOC services was evaluated using the United Nations (UN) process indicators in more than forty countries between 1999 and 2003 [4], [6], [9]–[14]. The recommendation is to have a minimum of 5 health facilities proving EOC per 500 000 population, at least 1 of which should provide CEOC [6]. In general the results showed that the number of facilities designated to provide CEOC was adequate with an average of 1–4 CEOC facilities per 500 000 population even in African and Asian countries where MMR is still high [4], [12]–[15]. The number of facilities expected to provide BEOC was consistently insufficient across countries. For example, 65–100% of facilities in surveyed African countries expected to provide BEOC could not perform the seven signal functions of BEOC [4], [11], [12], [15], similarly 63–87% of designated BEOC facilities were not fully functional in countries surveyed in South Asia [4], [11]–[14]. Furthermore, while in some settings an adequate proportion of women delivered in health facilities. The met-need for emergency obstetric care and the population based caesarean section rate were below the recommended minimum levels. Another reported a population CS rate of <1% to 3% comparing this to the recommended minimum of 5–15% of all pregnancies [6], [9]–[15].

Since these earlier surveys, international agencies and donors have significantly increased funding for maternal and newborn health (MNCH) programs [1], [16], [17] to help accelerate progress towards achievement of the Millennium Development Goals (MDG) [6], [10], [14], [18]–[20]. MDG 4 aims to reduce child mortality, with a target of reducing child deaths by two-thirds between 1990 and 2015. MDG 5 aims to improve maternal health with a target of reducing the maternal mortality ratio (MMR) by 75% by 2015 [20]. Health systems strengthening programmes in MNCH have focused on the upgrading of health facilities and infrastructure, purchasing and distribution of essential equipment, strengthening of supply chains for essential drugs. [10], [14], [18]–[23]. A decade after the initial assessments of EOC we report on availability and quality of EOC in four African and two Asian countries which have poor scores on maternal and newborn health indicators (Table 2). We examine the availability, utilization and quality of EOC in hospitals and health centres providing maternal and newborn care at both BEOC and CEOC level and include estimates of population coverage.

**Table 2:** Maternal and neonatal health indicators by country.

Country (DHS year)	Total Population (millions) †	TFR ‡	CBR (per 1000 population)‡	Estimated deliveriesper year	Percent of births assistedby skilled healthpersonnel*	Maternal Mortality Ratio(per 100,000live births)*	Neonatal mortality rate (per 1,000 live births)*
Sierra Leone (2008)	5.7	5.1	31.5	179,439	43	970	49
Nigeria (2008)	154.7	5.7	40.6	6,281,993	39	840	39
Kenya (2008/09)	39.8	4.6	34.8	1,385,11	42	530	27
Malawi (2010)	15.3	5.7	39.2	598,326	56	510	30
Bangladesh (2010)	158.7	2.7	24.7	3,919,026	27	194	30
India (2005/06)	1,181.0	2.7	23.1	26,913,000	47	230	34

Abbreviations: **TFR:** Total Fertility Rate; **CBR:** Crude Birth Rate.

† Source: The World Bank, 2011 (www.worldbank.org).

* Source: WHO World Health Statistics, 2011 http://www.who.int_whosis_whostat-EN_WHS2011.

‡ Source: Macro International Inc, 2011. MEASURE DHS STATcompiler http://www.measuredhs.com

## Methods

Cross sectional surveys of health care facilities providing maternal and neonatal health services were conducted in India, Bangladesh, Nigeria, Sierra Leone, Kenya and Malawi between 2009 and 2011. The countries were purposively selected, as they were targeted for implementation of the Making it Happen (MiH) programme which aims to increase quality and availability and quality of EOC. The state, province or district surveyed was selected and approved by the Ministry of Health of each country. For all countries general information about the state, province or districts in which surveys were conducted was obtained from the country’s latest Demographic and Health Survey (DHS), District Medical Officers in post and the District Health Management Information System (HMIS). This included: size of population, a list of all health facilities providing maternity services and maternal and newborn health indicators.

In Kenya, health services are organized into 6 levels; in ascending order with regard to level of care: dispensaries, health centres, sub-district hospitals, level five district hospitals, provincial government hospitals (PGHs) and teaching hospitals. All hospitals are expected to provide CEOC while health centres are expected to offer BEOC. All 10 PGHs and all facilities in six out of 12 districts in Nyanza Province were selected to participate in the surveys (Siaya, Kisumu West, Kuria, Migori, Homa Bay and Suba). These districts had a total population of 1.9 million. All 102 health facilities providing maternity services regardless of ownership status in the selected districts were included in the survey, giving a total of 112 surveyed facilities (hospitals and health centres) in Kenya.

In Nigeria, the health care system which includes teaching hospitals at the top followed by federal hospitals, general public hospitals, primary health care facilities (PHC), community health centres (CHCs) and health clinics/dispensaries at the lowest level. PHCs with maternity services are expected to function as BEOC facilities while CEOC should be available at all hospitals. Three states (Katsina, Zamfara, Yobe) situated in Northern Nigeria were selected by the Ministry of Health. All 51 hospitals (CEOC) in these states were assessed. This was followed by opportunistic selection of 8 of a total of 65 districts for an assessment of all other facilities providing maternity services. (Daure, Baure and Zango in Katsina state, Bursari, Geidam and Yunusari in Yobe state and Maru and Bungundu in Zamfara state) covering an estimated population of 12.1 million for the three states and 1.6 million for the 8 individual districts. All public, private or mission hospitals and health centres providing maternity services were assessed.

Sierra Leone has 3 administrative levels of health care; hospitals, community health centers (CHCs), and community health posts (CHPs). CHCs are expected to offer BEOC while hospitals are expected to offer CEOC. Data was collected in nine of the 14 districts in Sierra Leone; (Western urban district, Port Loko, Tonkolili, Kambia, Bombali, Bo, Pujehun, Kenema, and Kailahun), selection of districts was opportunistic. All public, private, mission and military hospitals providing maternity services as well as the health centers expected to be providing BEOC were assessed in each district. A total of 62 health facilities were assessed; 45 community health centres expected to provide BEOC and 16 hospitals expected to provide CEOC covering a total population of 4.4 million people.

In Malawi, four out of 12 districts in the Southern region were included in the survey (Mangochi, Machinga, Phalombe and Mulanje). All 8 hospitals in these 4 districts regardless of ownership and all health centres expected to provide BEOC (n = 31), covering a total population of 1.9 million were assessed. In Malawi, hospitals are expected to provide CEOC. Dispensaries which are the lowest level of care are not expected to provide maternity services.

In Bangladesh, the public health delivery system is organized into 4 levels; including medical college hospitals, followed by district level facilities (district hospitals (DH) and Maternal and Child Welfare Centres (MCWC), sub district facilities (Upazila Health Complexes UHC) and at the lower level are union level facilities (Family Welfare Centres FWCs, basic health units (BHUs) and rural health centers). Hospitals and MCWCs are expected to provide CEOC, while UHCs can be either a potential CEOC or BEOC depending on designation by the government. A selected number of FWCs are designated to provide BEOC, while basic health units (BHUs) and rural health centers are non-EmOC facilities. The Ministry of Health and Family Welfare selected four out of five divisions to be included in the survey. IIn each division, one district was identified (opportunistic) to participate in the study (Thakurgaon, Jamalpur, Maulvibazar and Narail). Only public health facilities were assessed in Bangladesh. All government Hospitals (DHs) and Maternal and Child Welfare Centres (MCWCs) providing EOC at the district level and all Upazilla Health Complexes (UHC) at sub-district level were assessed in the four districts. In total, 25 facilities were assessed; 8 district hospitals or Maternity and Child Welfare Centres and 17 Upazila Health Complexes covering a total population of 5.7 million people.

The Ministry of Health and Family Welfare in India has identified 7 states which have poorer health indicator scores than the national average. These states are referred to as ‘high focus states’ [24]. Four out of seven high focus states were included in the surveys (Madhya Pradesh, Bihar, Orissa and Chhattisgarh). One government district hospital and three hospitals or health centres were assessed in each of nine surveyed districts including; four districts (Tikangarh, Barwani, Jhabua and Anuppur) in Madhya Pradesh, two in Orissa (Angul and Kandhamal), two in Chhattisgarh (Raigarh and Rajnandangaon) and one in Bihar (Purnea) covering a total population in the surveyed districts of 11.7 million. In India as only a selection of public facilities was assessed per district, it was not possible to assess population level coverage.

All health facilities identified in each setting and included in the survey were visited by a research team composed of in-country data collectors and a member of the LSTM research team. At the facility, managers and clinical leads of the health centres were interviewed, registers were checked for the number of deliveries, stillbirths, women with obstetric complications and caesarean sections performed. The availability of equipment and drugs was also assessed by direct observation.

All information was collected using a pre-designed Rapid Assessment Tool (questionnaire) based on the UN EOC Assessment Manual and criteria [6]. In each country, data collection teams trained with each country team led by one or more researchers based at the Maternal and Newborn Health Unit at the Liverpool School of Tropical Medicine who also actively participated in facility visits and assessments. All data collection teams were trained for 2 days in-each country, in the use of the Rapid Assessment tool. Information on availability of signal functions, number of deliveries, identified obstetric complications, maternal deaths and stillbirths was collected for the period of three months prior to the facility visit [6].

Data was entered and analysed using the SPSS statistical software, version 18.0 (SPSS, Chicago, IL, USA). For assessment of availability of CEOC and BEOC signal functions all the facilities surveyed were included in the analysis. For population level assessments only health facilities specific to the 31 districts were included. Population measures for the availability and utilization of EOC were calculated based on the UN Process Indicators [6]. Proportions were calculated for different indicators and used to summarize the data.

### Ethical Approval

Permission to conduct the surveys and facility assessments was obtained from the Heads of Reproductive Health Unit, Ministry of Health of each respective country. The Provincial, State or Regional Medical Officers were informed in writing by the Ministries of Health. They in turn informed the respective District Health Management Teams and District Medical Officers. The district offices facilitated access to all health facilities. Health facility staff were informed about the surveys in writing and written informed consent was obtained from all health care workers who participated in the assessments.

## Results

A total of 378 health facilities were evaluated, 218 expected to be functioning as Basic Emergency Obstetric Care facilities (BEOC) and 160 as Comprehensive Emergency Obstetric Care facilities (CEOC). (Table 3).

**Table 3:** Minimum recommended number of health facilities expected to provide Emergency Obstetric Care (EmOC), number of health facilities available and number providing required signal functions for Basic and Comprehensive EOC by country.

Country	Total populationfor survey area	Minimum recommended number of CEOC facilities (1 per 500,000population)	Minimum recommended number of BEOC facilities (4 per 500,000population)	Total number of facilities available and surveyed	Number and proportion ofCEOC facilitiesproviding 9 signalfunctions	Number and proportion of BEOC facilities providing 7 signal functions
**Nigeria** * All hospitalsin 3 states	12,104,109	24	-	51	2/51 (4%)	-
**Nigeria** All facilities withmaternity care in8 districts	1,631,556	3	13	55	0/8 (0%)	1/47 (2%)
**Sierra Leone** All facilitiesin 9 districts	4,406,824	9	35	62	7/17 (41%)	0/45 (0%)
**Kenya** * All ProvincialGeneral hospitals	NA	NA	NA	10	5/10 (50%)	NA
**Kenya** All facilities withmaternity care in 6districts	1,955,034	4	16	102	3/26 (12%)	0/76 (0%)
**Malawi** All hospitals and HCexpected to provideBEOC in 4 districts	1,972,536	4	16	39	8/8 (100%)	2/31 (6%)
**Bangladesh** All publicDH, MCWCs and UHCsin 4 districts	5,764,539	12	46	25	3/19 (16%)	0/6 (0%)
**India** * Selected facilitiesin 4 high focus states	NA	NA	NA	34	9/21 (43%)	2/13 (15%)
**Total**	27, 834,598	56	126	**378**	**37/160 (23.1%)**	**5/218 (2.3%)**

* not included in district level population coverage estimates.

HC: Health Centers.

DH: District hospitals; MCWC: Maternal and Child Welfare Centres; UHC: Upazila Health Complexes.

BEOC: Basic EOC facility; CEOC: Comprehensive EOC facility.

### Availability of Facilities Providing EOC Services

Information to assess population coverage was available for 8 districts in Northern Nigeria, 6 districts in Kenya, 9 districts in Sierra Leone, 4 districts in Malawi and 4 districts in Bangladesh. The total population for these 31 districts is estimated to be 15.7 million.

Applying UN recommendations for minimum levels: for a population of 15.7 million the minimum required number of facilities providing EOC would be 158; out of those 32 providing CEOC and 126 providing BEOC.

The total number of health facilities in place in the 31 districts were more than sufficient to meet the UN minimum coverage levels for the population size for each country setting with a total of 283 health facilities in place; 205 designated to provide BEOC (126 needed; ratio 1.6) and 78 designated to provide CEOC (32 needed; ratio 2.4). (Table 3).

### Availability of EOC Signal Functions

With regard to ability to provide the required number of signal functions, only five (2.3%) of the 218 facilities expected to provide BEOC could provide all the seven signal functions required and 23.1% (37/160) facilities expected to provide CEOC could provide all the nine required signal functions. As facilities were considered fully functional if they were able to provide the 7 (BEOC) or 9 (CEOC) signal functions. None of the 31 districts included in the assessments met the minimum UN recommended coverage of 5 functioning EOC facilities per 500,000 population.

Of the 123 hospitals designated to be CEOC but unable to provide the nine signal functions needed, only one was able to provide the seven signal functions of BEOC. Taking individual countries into account, in Sierra Leone, Kenya and Bangladesh none of the designated BEOC were able to provide the full complement of seven signal functions. In the districts surveyed, the percentage of health facilities designated and fully functional as BEOC was 15% in India, 6% in Malawi and 2% in Nigeria. For CEOC only Malawi met the UN requirements with more than twice the number of fully functional CEOC facilities in place required for the population level. The proportion of CEOC facilities considered to be fully functional (all nine signal functions in place) was highest in Malawi (100%) and lowest in Nigeria (0%).

Availability of each individual EOC signal function differed between countries as well as between BEOC and CEOC level facilities. (Table 4). In all the countries, parenteral oxytocics, and antibiotics were the most frequently available EOC signal functions, with an average of 6–7 out of 10 facilities performing these functions. While more than 70% of facilities in Sierra Leone and Kenya were able to provide parenteral anticonvulsants, this was only 44% and 56% of facilities in the surveyed districts in Nigeria and Malawi respectively. Removal of retained products of conception and assisted vaginal delivery (AVD) were the least performed signal functions. Only 3–18% health facilities in the 4 African countries and 40% in Asian countries performed AVD. For the two additional signal functions of CEOC, blood transfusion was readily available; 7 and 9 out of 10 of health facilities in the two Asian and four African countries respectively offered the service. However, almost 20% of designated CEOC facilities were surveyed in Nigeria and Bangladesh and 40% in Kenya and India could not provide Caesarean Section.

**Table 4:** Availability of individual signal functions at BEOC and CEOC levels by country.

	Parenteral antibiotics	Parenteral oxytocics	Parenteral anticonvulsants	Manual removal of placenta	Removal of retainedproducts	Assisted vaginal delivery (vacuum extraction	Neonatal Resuscitation	Caesarean section	Blood transfusion
**Nigeria** All (106)	63% (67)	79% (84)	64% (68)	72% (76)	50% (53)	3% (3)			
CEmOC (59)	64% (38)	93% (55)	86% (51)	91% (54)	76% (45)	3% (2)			
BEOC (47)	62% (29)	62% (29)	36% (17)	47% (22)	17% (8)	2% (1)		81% (48)	90% (53)
**Sierra Leone** All (62)	92% (57)	92% (57)	73% (45)	50% (31)	26% (16)	18% (11)	65% (40)		
CEOC (17)	88% (15)	94% (16)	100% (17)	82% (14)	82% (14)	53% (9)	88% (15)		
BEOC (45)	93% (42)	91% (41)	62% (28)	39% (17)	4% (2)	4% (2)	56% (25)	88% (15)	88% (15)
**Kenya** All (112)	79% (89)	73% (82)	70% (78)	62% (70)	39% (44)	15% (17)			
CEOC (36)	89% (32)	94% (34)	89% (32)	89% (32)	67% (24)	31% (11)			
BEOC (76)	75% (57)	63% (48)	61% (46)	50% (38)	26% (20)	8% (6)		61% (22)	86% (31)
**Malawi** All (39)	62% (24)	97% (38)	56% (22)	46% (18)	46% (18)	31% (12)	31% (8/26) *		
CEOC (8)	100% (8)	100% (8)	100% (8)	100% (8)	100% (8)	100% (8)	100% (4/4) *		
BEOC (31)	52% (16)	97% (30)	45% (14)	32% (10)	32% (10)	13% (4)	18% (4/22)*	100% (8)	100% (8)
**Bangladesh** All (25)	100% (25)	96% (24)	68% (17)	80% (20)	44% (11)	40% (10)	68% (17)		
CEOC (19)	100% (19)	100% (19)	68% (13)	89% (17)	37% (7)	32% (6)	68% (13)		
BEOC (6)	100% (6)	83% (5)	67% (4)	50% (3)	67% (4)	67% (4)	67% (4)	79% (15)	68% (13)
**India** All (34)	100% (34)	94% (32)	65% (22)	62% (21)	53% (18)	38% (13)	82% (28)		
CEOC (21)	100% (21)	100% (21)	76% (16)	76% (16)	76% (16)	48% (10)	81% (17)		
BEOC (13)	100% (13)	85% (11)	46% (6)	38% (5)	15% (2)	23% (3)	85% (11)	62% (13)	67% (14)
**Mean All** (n = 378)	**78.3%** (296)	**83.9%** (317)	**66.7%** (152)	**62.4%** (236)	**42.3%** (160)	**17.5%** (66)	**65.6%** (103/157)		
**CEOC** (n = 160)	83.1% (133)	95.6% (153)	85.6% (137)	88.1% (141)	71.2% (114	28.8% (46)	78.7%(59/75)		
**BEOC** (n = 218)	74.8% (163)	75.2% (164)	52.8% (115)	43.6% (95)	)21.1% (46)	9.2% (20)	51.2%(44/86)	**75.6%** (121)	**83.7%** (134)

* Newborn resuscitation data only available for Mangochi district with 26 facilities.

**Figure 1:**
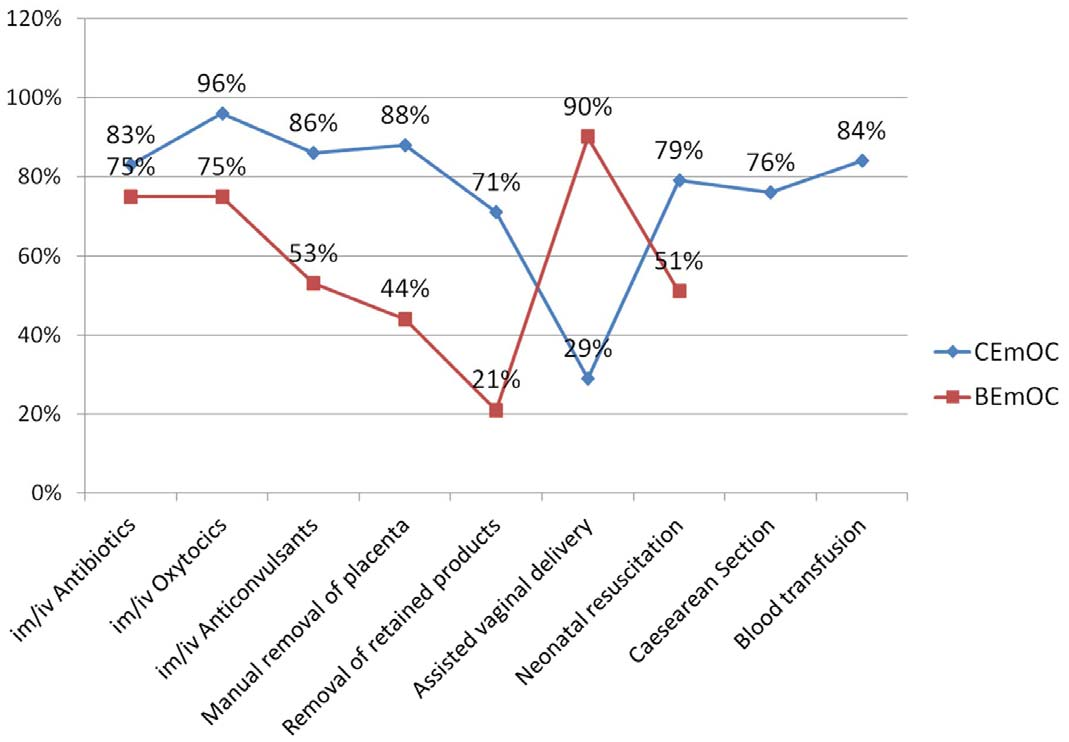
Signal functions available at Basic and Comprehensive Emergency Obstetric Care (EmOC) facilities (CEOC = 160; BEOC = 218). Abbreviations: IM, intramuscular; IV, intravenous.

Within countries there was marked disparity in availability of signal functions between CEOC and BEOC facilities, (Figure 1 & Table 3). Overall, signal functions were less available at BEOC compared to CEOC level. Removal of retained products of conception using Manual Vacuum Aspiration (MVA) for example was available in 76% of CEOC and 17% of BEOC facilities in Nigeria, in 82% and 4% of facilities in Sierra Leone, in 100% and 32% of facilities in Malawi and in 76% and 15% of health facilities in India respectively. AVD was equally not performed in CEOC (3%) and BEOC (2%) facilities in Nigeria. In other countries 31–100% of CEOC facilities were performing AVD compared to 4–23% of the BEOC facilities respectively.

### Uptake of EOC Services and Maternal and Newborn Health Outcomes

The proportion of expected births which occurred in health facilities in principle providing EOC in 31 districts across five countries ranged from 9.9% to 47.5%. (Table 5).

**Table 5:** Utilization and quality of EOC services including Maternal and newborn health outcomes for 31 districts by country.

Country (number of districts included in survey)	Kenya (6)	Nigeria (8)	Sierra Leone (9)	Malawi (4)	Bangladesh (4)
Population	1,955,034	1,631,556	4,406,824	1,972,536	5,764,539
Expected number of births per year^1^	98,739	68,526	138,815	83,636	142,382
Recorded number of births in the assessed facilities per year	19,492	10,932	18,764	39,712	14,132
Proportion of expected births taking place in assessed facilities	19.7%	15.9%	13.5%	47.5%	9.9%
Number of women expected to have EOCcomplications per year 2	14,811	10,278	20,822	12,546	21,357
Number of EOC complications recorded in assessed facilities per year	960	1,504	7,284	3,800	2,596
Met need for EOC^3^	6.5%	14.6%	35.0%	30.3%	12.2%
Number of Caesarean Sections (CS) per year	844	388	2,520	2,996	3,216
Population based CS rate^4^	0.9%	0.6%	1.8%	3.6%	2.3%
Number of recorded maternal deaths per year inassessed facilities	40	140	216	132	52
Case Fatality Rate for obstetric complications^5^	4.2%	9.3%	3.0%	3.5%	2.0%
Facility based maternal mortality ratio (per 100 000births)^6^	205	1,280	1,151	332	368
Number of recorded still births per year	416	304	1252	740	696
Facility based still birth rate	2.1%	2.8%	6.8%	1.9%	4.9%

1 Calculated by multiplying total population.

* crude birth rate.

2 Estimated as 15% of all expected births in the population.

3 Number of women who were admitted to the facility with EmOC complication divided by expected EmOC complications.

4 Number of CS performed as % of expected births in population.

5 Number of maternal deaths as a proportion of number of women recorded to have EmOC complications.

6 Number of maternal deaths as a proportion of number of births in the facility.

Using an expected population need for EOC based on 15% of all births anticipated and based on numbers admitted with complications in all health facilities designated to provide EOC, the met need for EOC ranged from 6.5% to 35.0%. Similarly, district level population-based CS rates are low in all countries; 0.6% in northern Nigeria, 0.9% in Nyanza districts in Kenya, 1.8% across the 9 districts in Sierra Leone, 2.3% across four districts in Bangladesh and 3.6% for four districts in Malawi respectively. Facility based Case Fatality (CFR) rates are above 1% in all districts surveyed ranging from 2.0% Bangladesh to 9.3% in northern Nigeria. Stillbirth rates at facility level ranged from 1.9% in Malawi to 6.8% in Sierra Leone.

## Discussion

The results of this survey illustrate a continued lack of availability of a simple care package of life saving interventions known as Emergency Obstetric Care (EmOC) across 6 countries with medium to high levels of maternal mortality. Whereas the absolute number of facilities expected to provide CEOC per 500,000 population was more than sufficient, we found the quality of services offered to be inadequate with many of the health facilities unable to provide all nine signal functions of EOC. Only 1 in 4 of the hospitals designated as a CEOC facility could in fact provide this package of interventions. Malawi was the only country which met the requirement of 1 CEOC per 500,000 population. Previous studies in Malawi reported 1.4–1.7 facilities per population [14], [19], [22].

In contrast, the number of facilities designated to provide BEOC was smaller than potentially needed for the population (coverage 0–0.5 facilities per 500 000 population). This is a disappointing finding illustrating a lack of improvement in the availability of BEOC compared to earlier surveys [4], [12]–[15]. In Malawi BEOC per 500 000 population was 0.1 in 2000, 0.2 in 2006, 0.0 in 2008 and 0.5 in the current study [14], [19], [22]. Similar to our results, in 2008, Oyerinde et al (2011) also noted that none of the facilities in Sierra Leone were providing BEOC [21], while in Uganda and Kenya only 2% of designated BEOC were reported to be fully functional in 2007 and 2009 [18], [25].

It was noted that assisted vaginal delivery (vacuum extraction) and removal of retained products of conception (by Manual Vacuum Aspiration - MVA or Dilatation and Curettage (D&C) were the least available signal functions [11], [15], [18], [21], [25]. But even the signal functions requiring relatively little skills such as parenteral administration of an antibiotic, anticonvulsant and oxytocic are still not universally available at health facilities. Haemorrhage is the single most common cause of maternal death. The simple procedure of administration of an oxytocic at time of birth by a health care provider will reduce the risk of postpartum haemorrhage by up to 60% [26]. Pre-eclampsia and eclampsia are the second most common cause of maternal death globally, and availability and proper use of magnesium sulphate has the potential to avert up to 85% of pre- or eclampsia related-deaths and disability [1], [27]. Sepsis kills many women each year. Recognition of sepsis postpartum and administration of antibiotics as treatment or as prophylaxis when needed is vital to reduce maternal mortality [6].

In the districts surveyed, indications are that the utilization of EOC is also low. The proportion of expected births which take place in EOC facilities ranged between 9.9% and 47.5%. The estimated met need for emergency obstetric care was less than 35% in most settings illustrating that many women with obstetric complications do not currently access a health facility for care. Known factors contributing to this are out of pocket expenditure for women and their families, non functional referral systems and distance or non equitable distribution of health facilities [28]. However it is likely that the non availability of care is well recognised by the population and that this too will be a strong reason for non-uptake of EOC. For women with obstetric complications who do come to a health facility the quality of care is poor as evidenced by high maternal case fatality rates (2.0–9.3%) and stillbirth rates (1.9–6.8%). In addition, it is likely that lack of knowledge and skills among healthcare providers results in failure to recognise conditions and manage them appropriately [23].

The population based CS rate is an estimate of accessibility and utilization of EOC for women with complications, especially obstructed labour. The rates obtained were low in this study: 0.6–3.6%. There is some evidence that CS rates are slowly increasing; in Malawi an increase from 1.6% in 2000 to 3.6% reported in the current study; in Kenya an increase from 0.6% in 2003 to 0.9%; in Sierra Leone an increase from <1% in 2008 to 1.8% and in Bangladesh an increase from 1.3% observed in 2000 to 2.3% in this study [10], [14], [15], [21], [22]. Despite the increase, the rates are below the minimum recommended level of 5% therefore it must be assumed that CS as a life-saving surgical intervention is still not provided for many women who need it in these settings.

Many of the facilities in principle designated to provide BEOC are situated in the more rural areas and could, if made functional to provide at least BEOC, help bridge the disparity between the rural and urban populations with regard to availability of health care [28]. The non availability of signal functions is likely to be a function of a combination of factors including lack of competency and skills among health care providers, lack of availability of simple drugs and equipment or good management systems to ensure equipment is maintained and there are no ‘stock outs’ in the facility of the basic drugs needed for EOC (which can often be bought outside the health facility in the open market [14], [23], [25].

The need to strengthen the procurement and distribution chain for basic drugs and equipment and the need to improve skills of providers to ensure at least minimum coverage of EOC is in place cannot be overemphasized. Task shifting by upgrading mid-level health providers to offer obstetric signal functions such as caesarean section and assisted vaginal delivery is one of the solutions. Studies in Malawi, Mozambique, Tanzania and Uganda have reported equally proficient performance and outcomes of caesarean sections among clinical officers and medical doctors [29]–[31]. Improved legislation for midwives to be allowed to do MVA, MRP, and assisted vaginal delivery (vacuum or ventouse delivery) also improve availability of EOC signal functions. Introduction of audit, provided this is conducted in a non judgemental manner and feedback is given to health care providers is a very strong tool to improve performance [32], [33]. van den Akker *et al,* (2011) and Dumont *et al*, (2006) respectively showed that in a period of two to three years systemic audit and feedback in busy district hospitals helped to improve management of haemorrhage and ruptured uterus and reduced CFR due to these two conditions by 45–68% respectively [34], [35]. It is recommended that all cadres of providers who are involved in maternal care as well as members of the community should be part of the maternal audit team [34]–[36]. This will ensure feedback is shared and action is taken to improve the quality of care.

This study has a number of limitations. Poor record keeping and especially recognition and recording of women with obstetric complications and/or the procedures carried out to manage such patients was noted in many health care facilities [9], [14], [18], [21]. Data on the number of women with EOC complications are not currently routinely collected in most labour ward registry books – although the number of deliveries and number of CS are generally accurately recorded. This will affect estimates provided of the met need for EOC as well as Case Fatality Rates. With interventions that lead to improved recognition, management and recording of women requiring EOC it is likely that the estimates of met need for EOC will increase and CFR will reduce [9]. This requires further study. Secondly, in Bangladesh and India, we assessed only public health facilities. We are aware that in Asian countries, private health facilities may contribute to the provision of maternity services especially in urban areas [10], [37]. Thus our result of coverage and utilization may underestimate the reality on the ground. In Nigeria and India, the surveys were conducted on relatively under-performing states and districts. Results cannot be generalised to the whole country and in future studies of this nature should consider comparing both high performing and low performing areas to give a more complete picture of status of EOC in area country.

Four years from 2015, the majority of women in the surveyed countries still do not have access to life-saving interventions for obstetric complications and early newborn care. The availability of both Comprehensive and Basic Emergency Obstetric Care is still well below stipulated minimum UN coverage rates first recommended in 1997. The availability and quality of care at facility level needs to be improved in order to reduce the number of maternal and newborn deaths. While the population based rate for CS has slightly improved, this is still far below the recommended minimum level of 5%. In order to achieve MDG 5 there is an urgent need to rethink strategies to BEOC, ensure improved coverage and quality of EOC services.

### Ethics Statement

The Respondents, Local Authorities, Heads of Reproductive Health Units, Ministries of Health of each respective country and were informed about the purpose of the survey. The Ministries of Health granted written approval and provided the survey teams with letters of authority to present to the district offices which facilitated access to all health facilities. Health facility staff were informed about the surveys in writing and written informed consent was obtained from all health care workers who gave information about maternal and newborn data of the facility. We did not collect any data regarding individual/patient information.

In consultation with the ethical board of Liverpool School of Tropical Medicine (LSTM), it was agreed that the type of research conducted falls into the category of monitoring and evaluation of the health service/system and these data are already in the public domain. As it did not involve data collection from patients, it did not need specific ethical board review.
